# Mediating Effect of Team Effectiveness of the Nursing Unit on the Nursing Work Environment and Patient-Centered Nursing: A Cross-Sectional Study

**DOI:** 10.3390/healthcare13172080

**Published:** 2025-08-22

**Authors:** Se Young Kim, Young Ko

**Affiliations:** 1 Department of Nursing, Changwon National University, 20 Changwondaehak-ro, Uichang-gu, Changwon 51140, Republic of Korea; sarakimk@changwon.ac.kr; 2 Department of Nursing, College of Nursing, Gachon University, Incheon 21936, Republic of Korea; 3 Gachon Biomedical Research Institute, Gachon University Gil Medical Center, Incheon 21565, Republic of Korea

**Keywords:** team effectiveness, nursing unit, nursing work environment, patient-centered nursing

## Abstract

Background/Objectives: This study aimed to examine the mediating role of the team effectiveness of the nursing unit on the relationship between the nursing work environment and patient-centered nursing. Methods: This cross-sectional design enrolled 327 nurses working in general hospitals across South Korea. Data were collected using a structured questionnaire between July and October 2022. Mediation analysis was conducted using PROCESS Macro v3.5 for SPSS (Model 4), with bootstrap resampling (5000 samples) to assess the direct and indirect effects of the nursing work environment on patient-centered nursing. Results: The mean score for patient-centered nursing was 3.96 (maximum scored 5). Clinical experience, position, and hospital type were found to be significant general characteristics influencing patient-centered nursing. Both the nursing work environment (r = 0.522, *p* < 0.001) and the team effectiveness of the nursing unit (r = 0.569, *p* < 0.001) showed significant positive correlations with patient-centered nursing. Moreover, team effectiveness was found to mediate the relationship between the nursing work environment and patient-centered nursing (R^2^ = 0.39, F = 35.35, *p* < 0.001). Conclusions: These findings highlight the importance of organization-level strategies to enhance the nursing work environment and foster a team-based culture to improve patient-centered nursing.

## 1. Introduction

Patient-centeredness, a concept presented in the 2001 report of the Institute of Medicine, “Crossing the Quality Chasm,” is a key element in the quality of healthcare [1]. Patient-centered care improves the quality of healthcare by building positive relationships between healthcare providers and patients, improving communication and encouraging active patient participation. In patient-centered care, the role of nurses who care for patients 24 h a day is the most important, and patient-centered nursing provides professional nursing that focuses on the patient’s individual needs [2]. Patient-centered nursing care respects the patient’s values and preferences, actively involves them in the treatment decision-making process, and provides nursing care tailored to the context of the individual’s life [3]. Patient-centered nursing encompasses not only routine nursing in which nurses administer medication according to the prescription or intervention through the nursing process in clinical practice but also activities such as listening to and empathizing with the individual needs of patients, involving patients in nursing, and communication among multidisciplinary team members about patients [4]. Scientific evidence continues to suggest that patient-centered nursing goes beyond simply increasing positive patient experience [5] and influencing the quality of nursing care and job satisfaction [6]. 

McCormack and McCance [7] proposed a patient-centered nursing model based on the theory of person-centered nursing and the results of previous studies, and posited that patient-centered nursing is possible only when personal factors, the organizational environment, and the nursing performance process work synchronously. The realization of patient-centered nursing is closely related to the environment in which nurses work [8]. In organizations where the nursing work environment is supportive, autonomy is guaranteed, and if the workload is appropriately adjusted, nurses can spend more time interacting with patients [9] and provide more empathetic and individualized care [10]. However, a poor work environment increases burnout and intention to leave [11], which can lead to lower patient safety and deterioration in the quality of nursing [12]. Therefore, to ensure the sustainability of patient-centered nursing, it is essential to establish a structural foundation that enables nurses to work in a safe and professionally satisfying environment.

The nursing unit is a core work unit within a healthcare organization that provides various nursing services such as direct nursing, nursing support, and communication to a certain number of patients, and is the site where team-based collaboration actually takes place [13]. According to previous studies, nurses’ perceptions of teamwork positively influence the promotion of patient-centered nursing [14]. For effective patient-centered nursing practice, teamwork based on setting nursing goals, information sharing, leadership, feedback, mutual support, and smooth communication is essential [15].

Most theoretical models conceptualize team effectiveness as a comprehensive construct that extends beyond simple teamwork [16,17]. These models emphasize that team effectiveness encompasses a combination of structural inputs, such as team members’ competence and composition; process elements—such as leadership, cohesion, communication, nursing competence, and coordination; and outcome indicators, including job satisfaction, work performance, team viability, and productivity. For example, the Input–Process–Output model [16] and Integrated Team Effectiveness Model [17] highlight the multidimensional nature of team functioning, incorporating contextual, dynamic, and performance-related factors to evaluate team effectiveness more comprehensively. Recently, as the importance of patient safety has garnered attention, interest in team effectiveness has been increasing in the healthcare field, and the need to consider organizational characteristics such as organizational culture and team effectiveness in relation to patient-centered nursing has been raised [18]. 

Furthermore, the team effectiveness of the nursing unit may serve as a critical mediating factor in delivering patient-centered nursing care. Supportive and autonomy enhancing work environments facilitate open communication, cooperative behavior, and effective leadership among nurses, which in turn strengthen team effectiveness [19]. This team effectiveness forms the basis for nurses to actively respond to patients’ needs and provide coordinated care [20]. In other words, the influence of the nursing work environment on patient-centered nursing may be indirectly mediated or amplified by team effectiveness. To enhance patient-centered nursing, it is essential to improve the quality of the nursing work environment and to foster and sustain a team-based nursing culture. Therefore, this study aimed to identify the level of patient-centered nursing and to explore the role of the team effectiveness of the nursing unit in the relationship between the nursing work environment and patient-centered nursing among nurses who work in a nursing unit at a general hospital. We hypothesized that (1) patient-centered nursing is positively correlated with the nursing work environment and team effectiveness of the nursing unit and (2) the team effectiveness of the nursing unit plays a mediating role in the relationship between the nursing work environment and patient-centered nursing.

## 2. Materials and Methods

### 2.1. Study Design and Participants

This study employed a cross-sectional survey design to investigate the role of nursing unit team effectiveness in the relationship between the nursing work environment and patient-centered nursing. Participants were registered nurses working in six general hospitals in South Korea who were recruited through convenience sampling. The inclusion criteria were nurses working in the ward who understood the purpose and method of this study and voluntarily agreed to participate in the survey. The exclusion criteria were nurses working in special care units or outpatient department.

To ensure sufficient statistical power to examine the factors influencing patient-centered nursing, an a priori power analysis was conducted using G*Power 3.1.9.7. Based on previous studies reporting the correlations between the nursing work environment and person-centered nursing (r = 0.19) [21], and between teamwork and person-centered nursing (r = 0.21) [15], the effect size was estimated at f^2^ = 0.087. With a significance level of 0.05, statistical power of 0.95, and 12 predictor variables, the minimum required sample size for multiple regression analysis was calculated to be 308. A total of 327 questionnaires from 55 nursing units were collected and included in the final analysis. The inclusion of data from 327 participants demonstrates the adequacy of the sample and confirms its feasibility for addressing the research objectives.

### 2.2. Measures

#### 2.2.1. Patient-Centered Nursing

The patient-centered nursing activities sub-domain of the patient-centered nursing culture scale developed by Shin and Yoon [22] was used to measure patient-centered nursing in this study. This scale was developed specifically within the Korean healthcare context, reflecting culturally relevant aspects of patient-centered care. This tool consists 11 items, including support for patient participation, patient respect, listening, detailed explanation, emotional support, and sincere nursing behavior. The score is assigned on a 1–5 point scale; a higher average score implies greater patient-centered nursing performance. Its validity was established through exploratory and confirmatory factor analyses, and it demonstrated high internal consistency (Cronbach’s α = 0.90) at the time of development [22].

#### 2.2.2. Team Effectiveness of the Nursing Unit

The Team Effectiveness Scale for Nursing Units (TES-NU), originally developed by Kim and Kim [23] based on the Integrated Team Effectiveness Model [17,24] and subsequently refined by Kim et al. [25], was employed in this study. The refined instrument comprises 22 items across five subdomains: head nurse leadership, job satisfaction, cohesion, work performance, and nursing competence. Each item is rated on a 5-point Likert scale, with higher average scores reflecting greater team effectiveness within the nursing unit. The reliability of the tool, as measured by Cronbach’s alpha, was 0.94 at the time of development [23] and 0.92 following refinement [25].

#### 2.2.3. Nursing Working Environment 

Based on the organizational characteristics of magnet hospitals with good working environments in the United States, Kramer and Hafner [26] developed the Nursing Work Index (NWI), which was modified by Aiken and Patrician [27] to develop the Revised Nursing Work Index (NWI-R). Lake [28] modified it again to develop the Practice Environment Sale of Nursing Work Index (PES-NWI). The Korean version of the PES-NWI developed by Cho et al. [29] consists of 29 items graded on a scale of 1 to 4, and the score is calculated by computing the average value of the entire or sub-domains. The reliability of the tool at the time of development, as measuring using Cronbach’s alpha, was 0.93 [29].

#### 2.2.4. Covariates

We considered covariates such as sex, age, marital status, education, clinical experience, the work experience of the current unit, position, type of nursing unit, type of hospital, and ward bed-to-nurse ratio. The ward bed-to-nurse ratio was calculated as the number of ward beds divided by the number of ward full-time nurses [30]. 

### 2.3. Data Collection

Data were collected between July and October 2022. This study was conducted with the approval of the institutional review board (IRB no: 7001066-202205-HR-029). The researchers visited six general hospitals and explained the purpose and methods of the study. The hospitals selected nursing units that would participate in the survey using the convenience sampling method. The researchers visited the nursing units in person and distributed six questionnaires to each unit. The nurses (from each unit), who wanted to participate in the study, read the purpose and methods of the research, voluntarily agreed to participate, and filled out the questionnaire. The participants placed the completed questionnaire in the collection envelope installed in the nursing unit, which was collected by the researchers. 

### 2.4. Statistical Analysis

We analyzed the data using SPSS, version 26.0 (IBM Corporation, Armonk, NY, USA). Participants’ general characteristics, patient-centered nursing, TES-NU, and PES-NWI (Korean version) were presented as frequencies and percentages, and averages and standard deviations. Pearson’s correlation was used to analyze the relationships between the patient-centered nursing, team effectiveness of the nursing unit, and nursing work environment. Independent sample t-tests and one-way analysis of variance (ANOVA) were used to evaluate the associations between patient-centered nursing and categorical variables among the general characteristics. Pearson’s correlation was used to analyze the relationships between the ward bed-to-nurse ratio and patient-centered nursing. Finally, a mediation analysis was conducted using Model 4 of the PROCESS Macro for SPSS (Version 3.5), which estimates direct and indirect effects through ordinary least squares regression. This approach is suitable for testing simple mediation models and incorporates bootstrapping procedures to assess the statistical significance of indirect effects. In this study, the direct and indirect effects of the nursing work environment on patient-centered nursing were examined using 5000 bootstrap samples [31].

We entered the independent variables that were significantly related to the dependent variable in the univariate analysis into the mediating model. *p*-values < 0.05 were considered significant. 

## 3. Results

### 3.1. Participant Characteristics 

Of the 327 participants, 16 were male and 311 were female. The participants’ average age was 29.23 (±5.04) years. Approximately 80% were single, and 69.7% had a bachelor’s degree or higher. Overall, 91.7% of the participants were staff nurses and 8.3% were charge nurses. Regarding the type of institution, 61.5% worked at general hospitals and 38.5% at tertiary general hospitals. The patient-centered nursing activities score was 3.96 (maximum score: 5 points), the TES-NU (team effectiveness of the nursing unit) was 3.06 (0.35), and the PES-NWI (nurse work environment, maximum score: 5 points) was 2.75 (0.41). The participants’ general characteristics are presented in Table 1.

### 3.2. Correlation Analysis of Patient-Centered Nursing, Nursing Unit Team Effectiveness, and Nursing Work Environment

Higher levels of patient-centered nursing were associated with a better nursing work environment (r = 0.522, *p* < 0.001) and higher team effectiveness of the nursing unit (r = 0.569, *p* < 0.001).

### 3.3. Relationships Between General Characteristics and Patient-Centered Nursing

In the univariate analysis, clinical experience, position, and the type of hospital were general characteristics associated with patient-centered nursing (Table 2). A higher level of ward bed-to-nurse ratio was not associated with patient-centered nursing (r = −0.058, *p* = 0.292). Variables that were statistically significant in the univariate analyses were included in multivariable analysis. 

### 3.4. Mediating Effects of Nursing Unit Team Effectiveness on Nursing Working Environment and Patient-Centered Nursing

**Figure 1:**
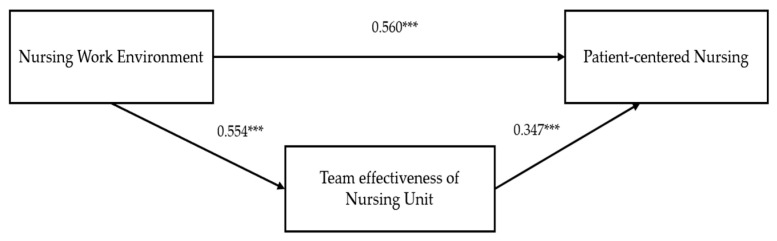
Mediating effect of team effectiveness of the nursing unit on the relationship between nursing work environment and patient-centered nursing. Conceptual model depicting the mediating effect of team effectiveness of nursing unit on the relationship between the nursing work environment and patient-centered nursing. All pathways were statistically significant (*** *p* < 0.001), and coefficients are based on regression analyses using PROCESS Macro (Model 4) with 5000 bootstrap samples. Control variables included clinical experience, position, and type of hospital.

Table 3 and Table 4 show the mediating effect of the team effectiveness of the nursing unit on the nursing working environment and patient-centered nursing, after controlling for the covariates. The nursing working environment exerted a significant direct effect on patient-centered nursing activities (β = 0.560, *p* < 0.001), with higher scores for the nursing working environment being associated with a higher level of patient-centered nursing. The indirect effect of the nursing work environment on patient-centered nursing, mediated via the team effectiveness of the nursing unit, was found to be statistically significant (β = 0.311, *p* < 0.001), indicating that the nursing work environment influenced the team effectiveness of the nursing unit (β = 0.554, *p* < 0.001), which in turn influenced patient-centered nursing (β = 0.347, *p* < 0.001). Generally, the total effect of the nursing work environment on patient-centered nursing was significant (β = 0.657, *p* < 0.001). The complete model results are presented in Figure 1.

## 4. Discussion

This study was conducted to investigate the level of patient-centered nursing of nurses working in nursing units in general hospitals and to confirm the role of nursing unit team effectiveness in the relationship between the nursing work environment and patient-centered nursing.

First, the average person-centered nursing activities score of the study participants was 3.96 (0.50) (maximum: 5 points), which was slightly higher than that of public hospital nurses in Korea [32] measured using another tool and similar to that of nurses working at a major university hospital in Finland [33]. In addition, the results of this study are similar to those of a study targeting nurses in seven European countries (3.87–4.25) [34]. Although scores may vary across studies due to disparities in the demographic characteristics of the participants, most studies have reported that nurses perform patient-centered nursing activities at an intermediate or higher level. The consistent pattern of moderate to high performance supports the growing global emphasis on patient-centered care as a core nursing value.

In this study, patient-centered nursing activities were higher for nurses who worked for less than 3 years compared to those who worked for more than 5 years, in staff nurses compared to charge nurses, and in tertiary general hospitals. These results are consistent with those of previous research that found no relationship between the socioeconomic characteristics of nurses and patient-centered nursing [35]. Results based on nurses’ work experience and work environment, such as the type of nursing unit or hospital, vary across studies [32,36]. 

The results of this study showed that nurses in tertiary general hospitals performed more patient-centered nursing than nurses in general hospitals. In Korea, a tertiary general hospital is a government-designated medical institution that provides advanced and high-complexity care; requires a higher level of staffing and medical resources; and serves as a central hub for education, research, and the national healthcare delivery system [37].

Tertiary general hospitals have a higher proportion of general nursing care units than general hospitals. In addition, the general nursing care unit of tertiary general hospitals requires greater expertise from nurses because these institutions admit patients with high disease severity and employ relatively more nursing staff as well as nurse assistants than general hospitals [30], to ensure a more systematic and individualized nursing approach. These results were also confirmed in a previous study that found that tertiary general hospitals had higher patient-centered experience scores than general hospitals [38].

The results of this study are consistent with those of a previous study that found that nurses working in comprehensive nursing care units exhibited relatively lower levels of patient-centeredness than those in general nursing units, due to heightened concerns and responsibilities related to patient safety incidents and reduced opportunities for patient engagement [14]. In Korea, comprehensive nursing care units provide integrated nursing services in which the nursing team directly cares for patients without caregiver assistance, unlike general nursing units where family members or private caregivers often participate in patient care [39]. Notably, the finding that comprehensive nursing care units exhibited lower patient-centered nursing scores despite employing twice as many nurses as general nursing units suggests a need for targeted improvements to enhance patient-centered nursing in general hospitals.

The relationship between clinical experience and patient-centered nursing is inconsistent across studies [36,40]. Experienced nurses display high levels of competence in understanding individual patient needs, empathic communication, and multidisciplinary collaboration [40]. Increasing clinical experience improves the professionalism of nurses, enabling them to practice more person-centered nursing [41]. Based on the results of these two studies, it is reasonable to assume that clinical experience is related to patient-centered nursing. However, previous studies have shown that excessive workload, lack of personnel, and unreasonable social and institutional demands are factors impeding patient-centered nursing practice [40], while time constraints are barriers to patient participation [4,42]. Organizational factors, rather than individual nurse-related factors, may exert a greater influence on patient-centered nursing. This aligns with the results of the current study, which showed that the significance of clinical experience disappeared upon the introduction of the nursing work environment. Since clinical experience was not analyzed independently, it is difficult to confirm a clear relationship, necessitating a future study to confirm the detailed relationship between work competency that improves with experience accumulation and patient-centered nursing.

Second, the results of this study, that is, the positive correlation of both the nursing work environment and nursing unit team effectiveness with patient-centered nursing, are consistent with those of previous studies [9,10,14]. Safe and guaranteed autonomy in the work environment, an appropriate workload, and support from colleagues and leaders create an environment wherein nurses can focus more on patients and deliver empathic and individualized care [9]. Similar to the relationship between teamwork and patient-centered nursing [14], the effectiveness of the nursing unit teams was confirmed to be related to patient-centered nursing.

Nursing unit team effectiveness covers sub-domains such as nurse manager leadership, job satisfaction, team cohesion, job performance, and nurse competency. Although not explicitly visualized in the results of this study, multiple regression analysis found that job satisfaction and team cohesion were most strongly associated with patient-centered nursing. These findings align with previous research indicating that higher job satisfaction is linked to lower burnout among nurses [43], and that reduced burnout enhances nurses’ capacity to engage in meaningful communication and build empathic relationships with patients [44]. Moreover, when trust and collaboration are well established within a nursing unit, team members are more likely to share information and engage in mutual problem-solving, ultimately contributing to more consistent and coordinated patient care [14]. In this context, strong team cohesion fosters a collective sense of responsibility, where individual performance is seen as a reflection of team success, thereby promoting team-based patient-centered care. High-performing nursing units, therefore, tend to cultivate a work climate characterized by mutual trust, respect, and collaboration, which supports integrated and consistent care through active information sharing, consideration of patient needs and preferences, and collaborative decision-making. While this study focused on the structural and relational aspects of team effectiveness at the nursing unit such as cohesion and job satisfaction, it is important to consider that team functioning is embedded within a broader organizational context. Factors such as organizational culture, power dynamics, and conflict management strategies may significantly influence team effectiveness. Future studies should investigate how these organizational factors interact with the team effectiveness of nursing unit to influence the delivery of patient-centered care.

Finally, this study confirmed the mediating effect of nursing unit team effectiveness on the nursing work environment and patient-centered nursing. A supportive and positive work environment can enhance team cohesion by promoting trust, communication, and cooperation among nurses, and improved team dynamics can lead to more collaborative nursing, where nurses discuss and coordinate patient conditions and needs together, ultimately leading to the more active provision of patient-centered nursing. Additionally, creating an atmosphere for realizing patient-centered values at the nursing unit team level can also exert a positive effect on the behavior of individual nurses.

These results are consistent with the Input–Process–Output and Integrated Team Effectiveness Models, which conceptualize team effectiveness as emerging from the interaction of structural inputs (e.g., work environment), team processes (e.g., communication, cohesion), and outcomes (e.g., job satisfaction, work performance) [16,17]. The present study supports these frameworks by demonstrating that a supportive nursing work environment (input) enhances team effectiveness (process), which in turn promotes patient-centered nursing (outcome). This result supports the theoretical framework and highlights the practical relevance of these models in explaining how nursing team dynamics contribute to care quality.

Overall, the results of this study suggest that patient-centered nursing is not simply influenced by the competency or attitude of individual nurses but is a collective nursing practice influenced by structural and cultural factors at the organizational level. Therefore, to promote patient-centered nursing, improvement of the organization-level work environment and team-based culture creation must be implemented. 

This study is significant as it identifies the mediating role of nursing unit team effectiveness in the relationship between the nursing work environment and patient-centered nursing. It also emphasizes the need for organizational-level strategies to foster team-based, patient-centered care, and provides directions for future interventions.

However, this study has some limitations. First, due to the cross-sectional design of this study, the temporal sequence among the nursing work environment, the team effectiveness of the nursing unit, and patient-centered nursing could not be determined. As such, the observed associations should be interpreted as correlational rather than causal. Future research employing longitudinal or experimental designs is needed to clarify the directionality and establish causal pathways between these variables. Another limitation of this study lies in the use of convenience sampling. Although it allowed for efficient recruitment across multiple hospitals and nursing units, it inherently limits the representativeness of the sample. Nurses who voluntarily chose to participate may differ systematically from those who did not, potentially leading to self-selection bias. Moreover, the sample may not fully reflect the institutional diversity of nursing units across Korea, particularly in terms of geographical location, hospital size, or staffing patterns. Caution is needed when generalizing research results. Lastly, all variables were measured using self-report questionnaires, which may be subject to social desirability bias and may not fully capture actual behaviors or interactions within nursing units.

## 5. Conclusions

This study confirmed that both the nursing work environment and nursing unit team effectiveness are positively associated with patient-centered nursing. Notably, team effectiveness—particularly job satisfaction and team cohesion—mediated the relationship between the work environment and patient-centered care. These findings suggest that patient-centered nursing is influenced not only by individual nurse characteristics but also by organizational structures and team dynamics. Therefore, to promote patient-centered care, it is essential to improve the nursing work environment and cultivate a collaborative team culture within nursing units. Despite the limitations arising from the cross-sectional design and convenience sampling, this study provides foundational evidence for organization-level interventions aimed at enhancing patient-centered nursing.

## Figures and Tables

**Table 1:** General characteristics, patient-centered nursing, team effectiveness of the nursing unit, and nursing working environments (*N* = 327).

Variables	Category	*n* (%)	M (SD)
Sex, *n* (%)	Male	16 (4.9)	
	Female	311 (95.1)	
Age (years), *n* (%)	≤29	229 (70.0)	
	30–39	79 (24.2)	
	≥40	19 (8.8)	
Age (years), M (SD)			29.23 (5.04)
Marital status, *n* (%)	Not married	263 (80.4)	
	Married	64 (18.6)	
Education, *n* (%)	Associated degree	90 (30.3)	
	Bachelor’s degree	213 (65.1)	
	Master’s degree	15 (4.6)	
Clinical experience (years), *n* (%)	≤3	73 (22.3)	
	3–5	100 (30.6)	
	>5	154 (47.1)	
Clinical experience (years), M (SD)			6.19 (5.04)
Work experience in current unit (years), *n* (%)	≤1	57 (17.4)	
	1–3	101 (30.9)	
	>3	169 (51.7)	
Work experience in current unit (years), M (SD)			3.96 (3.56)
Position, *n* (%)	Staff nurse	300 (91.7)	
	Charge nurse	27 (8.3)	
Type of nursing unit	General nursing care unit	167 (51.1)	
	Comprehensive nursing care unit	160 (48.9)	
Type of hospital, *n* (%)	Tertiary general hospital	126 (38.5)	
	General hospital	201 (61.5)	
Ward bed-to-nurse ratio, M (SD), Range = [0.19, 3.85]			1.91 (0.77)
Patient-centered nursing activities score, M (SD) Range = [2.64, 5.00]			3.96 (0.50)
TES-NU, M (SD), Range = [1.87, 3.82]			3.06 (0.35)
PES-NWI-Korean version, M (SD), Range = [1.48, 3.90]			2.75 (0.41)

Note: TES-NU: Team Effectiveness Scale for Nursing Units, PES-NWI: Practice Environment Sale of Nursing Work Index.

**Table 2:** Univariable analyses of the general characteristics associated with patient-centered nursing (*N* = 327).

Variables	Category	M (SD)	t or F	*p*
Sex	Male	3.95 (0.50)	−1.60	0.111
	Female	4.15 (0.54)		
Age (years)	≤29	3.97 (0.48)	1.79	0.169
	30–39	3.88 (0.56)		
	≥40	4.10 (0.54)		
Marital status	Not married	3.95 (0.47)	−0.24	0.810
	Married	3.97 (0.61)		
Education	Associated degree	4.02 (0.53)	1.44	0.239
	Bachelor’s degree	4.10 (0.43)		
	Master’s degree	3.93 (0.48)		
Clinical experience (years) ^†^	≤3 ^a^	4.10 (0.43)	3.71	0.025
	3–5 ^b^	3.93 (0.48)		(a > c)
	>5 ^c^	3.91 (0.54)		
Work experience in current unit (years) ^†^	≤1 ^a^	3.98 (0.55)	1.67	0.191
	1–3 ^b^	4.02 (0.46)		
	>3 ^c^	3.91 (0.51)		
Position	Staff nurse	3.98 (0.49)	2.74	0.007
	Charge nurse	3.70 (0.58)		
Type of nursing unit	General nursing care unit	3.97 (0.50)	0.48	0.630
	Comprehensive nursing care unit	3.94 (0.40)		
Type of hospital	Tertiary general hospital	4.12 (0.52)	4.83	<0.001
	General hospital	3.86 (0.47)		

Note: ^†^ Significant difference in the post hoc test were determined using the Scheffe tests (subgroups ^a^, ^b^, or ^c^, *p* < 0.05).

**Table 3:** Mediating effect of team effectiveness of the nursing unit on the nursing work environment and patient-centered nursing (*N* = 327).

Variables	Patient-Centered Nursing (Y)				95% CI	
	β	SE	t	*p*	LLCI	ULCI
Team effectiveness of the nursing unit (M)	0.347	0.071	4.90	<0.001	0.207	0.486
Nursing work environment (X)	0.560	0.082	6.80	<0.001	0.398	0.722
R^2^	0.39					
F	35.35					
*p*	<0.001					

Note: Clinical experience, position, and type of hospital were controlled for this model; Y = dependent variable; CI: confidence interval; SE = standard error; LL = lower limit; UL = upper limit; M = mediator; and X = independent variable.

**Table 4:** Total, direct, and indirect effects of the nursing work environment on patient-centered nursing through team effectiveness of the nursing unit (*N* = 327).

Categories	Effect	SE	t	*p*	95% CI	
					LLCI	ULCI
Total effect	0.657	0.058	11.37	<0.001	0.544	0.771
Direct effect	0.347	0.071	4.90	<0.001	0.207	0.486
Indirect effect	0.311	0.053			0.208	0.419

Note: Clinical experience, position, and type of hospital were controlled for this model; SE = standard error; CI: confidence interval; LL = lower limit; and UL = upper limit, 5000 bootstrap iterations.

## Data Availability

The data that support the findings of this study are available from the corresponding author upon reasonable request.
